# EGTool: an evolutionary genome topology tool for multiscale comparative genomics

**DOI:** 10.1093/bioadv/vbag330

**Published:** 2026-11-02

**Authors:** Fatih Sarigol, Darrin T Schultz, Oleg Simakov

**Affiliations:** Department for Neurosciences and Developmental Biology, University of Vienna, Vienna 1030, Austria; Department for Neurosciences and Developmental Biology, University of Vienna, Vienna 1030, Austria; Department of Neurobiology, Stanford University, Stanford, CA 94305, United States; Department for Neurosciences and Developmental Biology, University of Vienna, Vienna 1030, Austria

## Abstract

**Summary:**

Increased phylogenomic sampling and availability of thousands of high-quality genome assemblies require tools to simultaneously analyze changes to both chromosomal and subchromosomal genome architecture. Recently, we proposed an evolutionary genome topology (EGT) approach to identify and associate genome structural changes with evolutionary transitions. In this Application Note, we provide a generalized implementation of this approach, which we call EGTool. EGTool compares genomes to each other (multigenome topology) or loci to each other across genomes (multilocus topology), accepts arbitrary positional and orthology inputs, works in base-pair or index coordinates, projects new genomes onto an existing embedding without refitting, and recovers the features that characterize any chosen region of the projection via inverse transform. A 510-genome, 1274-locus example is included to illustrate these functionalities and can be run on a laptop within 30 min.

**Availability and implementation:**

EGTool is implemented in Python (≥3.8) with dependencies on NumPy, pandas, umap-learn, matplotlib, NetworkX, and scikit-learn. It is installable via pip after cloning the repository. The optional ancestral linkage group detection step (egtool alg) requires R. A Perl script is provided for converting genome annotation files to the input .chrom format. Source code, example data, and tutorials are available at: https://bitbucket.org/viemet/egtool/src/main/

## 1 Introduction

The chromosome-scale genome assemblies now emerging for thousands of genomes across eukaryotic diversity unlock the study of genome architecture evolution across scales, from individual genes to whole chromosomes (Simakov and Kawashima 2017, Schultz and Simakov 2026). While quantification of genome organization, e.g. via synteny approaches, has been possible in many tools (Wang *et al.* 2012, Simakov *et al.* 2013, Schultz *et al.* 2023) already, these approaches were mainly limited to specific scales or thresholds of genomic organization (such as chromosomal homologies, microsyntenies, etc.). We recently introduced the evolutionary genome topology (EGT) approach, which uses pairwise ortholog distances to capture both chromosomal and subchromosomal fusion-with-mixing (FWM) events (Schultz *et al.* 2026, Schultz and Simakov 2026). This multiscale approach enables visualization of evolutionary trajectories across thousands of species, providing a new view on phylogenomic diversity, as well as enabling prediction of thousands of putative regulatory linkages that arose through FWM and local enhancer-promoter contact mixing (“entanglement”) processes (Simakov and Wagner 2025). At its core, EGT represents each genome, or each locus, as a vector of pairwise distances between orthologous features and embeds those vectors in two dimensions using uniform manifold approximation and projection (UMAP; McInnes *et al.* 2018) or t-distributed stochastic neighbor embedding (t-SNE; van der Maaten and Hinton 2008). Genomes with similar gene-spacing configurations therefore fall close together in the projection, forming trajectories that reflect accumulation of positionally constrained loci (e.g. via FWM at the chromosomal scale, Schultz *et al.* 2026). While earlier comparative genomics software has employed multidimensional scaling (Pelé *et al.* 2012), and more recent packages have used a variety of manifold learning techniques (Zhang *et al.* 2021), neither provides an integrated pipeline for topology-based genomic analysis.

Building on our previous EGT implementation (Schultz *et al.* 2026, Schultz and Simakov 2026), we developed EGTool, a new generalized implementation that provides a user-friendly interface alongside support for additional distance transformations and normalization schemes, as well as new genomic data projection choices and inverse transform computations. With EGTool, a broad range of users can compute EGT representations and identify the most important genomic feature associations across many genomes. The tool can aid phylogenomic classification, genomic novelty (e.g. regulatory constraint) identification, identification of unexplored or endangered genomic neighborhoods (e.g. for conservation genomics), as well as emerging research in predicting future evolutionary trajectories of genomic configurations in biodiversity and medical genomic applications (summarized in Schultz and Simakov 2026).

EGTool accepts a broad range of positional and orthology input formats, supports both real base-pair and index-based (e.g. the order of a gene along a chromosome) distance calculations, and implements multiple distance metrics. The tool constructs both multigenome topology (MGT) projections, where species are compared by their pairwise distances of orthologous loci, and multilocus topology (MLT) projections, where orthologous features are compared by their averaged distances across genomes (Schultz *et al.* 2026). In addition, EGTool provides inverse transform functionality to identify the characteristic genomic features underlying specific regions of the manifold.

## 2 Methods

### 2.1. Data input

EGTool is designed to work with a broad range of positional and orthology input formats and is readily adaptable to new data types as they become available. Orthologous features can be of any type (coding genes, noncoding elements, etc.). While orthology detection is not part of the toolkit, several available programs such as OrthoFinder (Emms and Kelly 2015) or mapping of known orthologous markers as done by ODP (Schultz *et al.* 2023) can be used. Any orthology information and in any species group (plants, animals, fungi, etc.) with chromosomal-scale genomic data can be used. As in previous work, we use a simple .chrom format that can be easily obtained from a typical .gff format via provided scripts. A script (def_alg.R, invoked by the “egtool alg” command) can be used to identify chromosomal homologies (ancestral linkage groups, ALGs) using Fisher’s exact test as described in previous studies (Putnam *et al.* 2007, Simakov *et al.* 2022).

### 2.2. Preparation of distance data files

Available .chrom and orthology information files are parsed by the prep_egt.py script (invoked by the “egtool prep” command) to calculate pairwise distances between all possible genomic feature combinations. The script takes the orthogroup information (Fig. 1), a list or a file of .chrom files (one .chrom file per species). Orthology information can be protein-coding genes (e.g. from OrthoFinder, Emms and Kelly 2015) or any other genomic feature, such as mapped locations of noncoding elements. In addition, the script can check for the minimum fraction of species in which a given pair of orthologous features is represented on a single chromosome. In the example dataset for 510 species, at least 90% of species were required to have chromosomal colocalization, resulting in 3113 pairwise entries.

**Figure 1:**
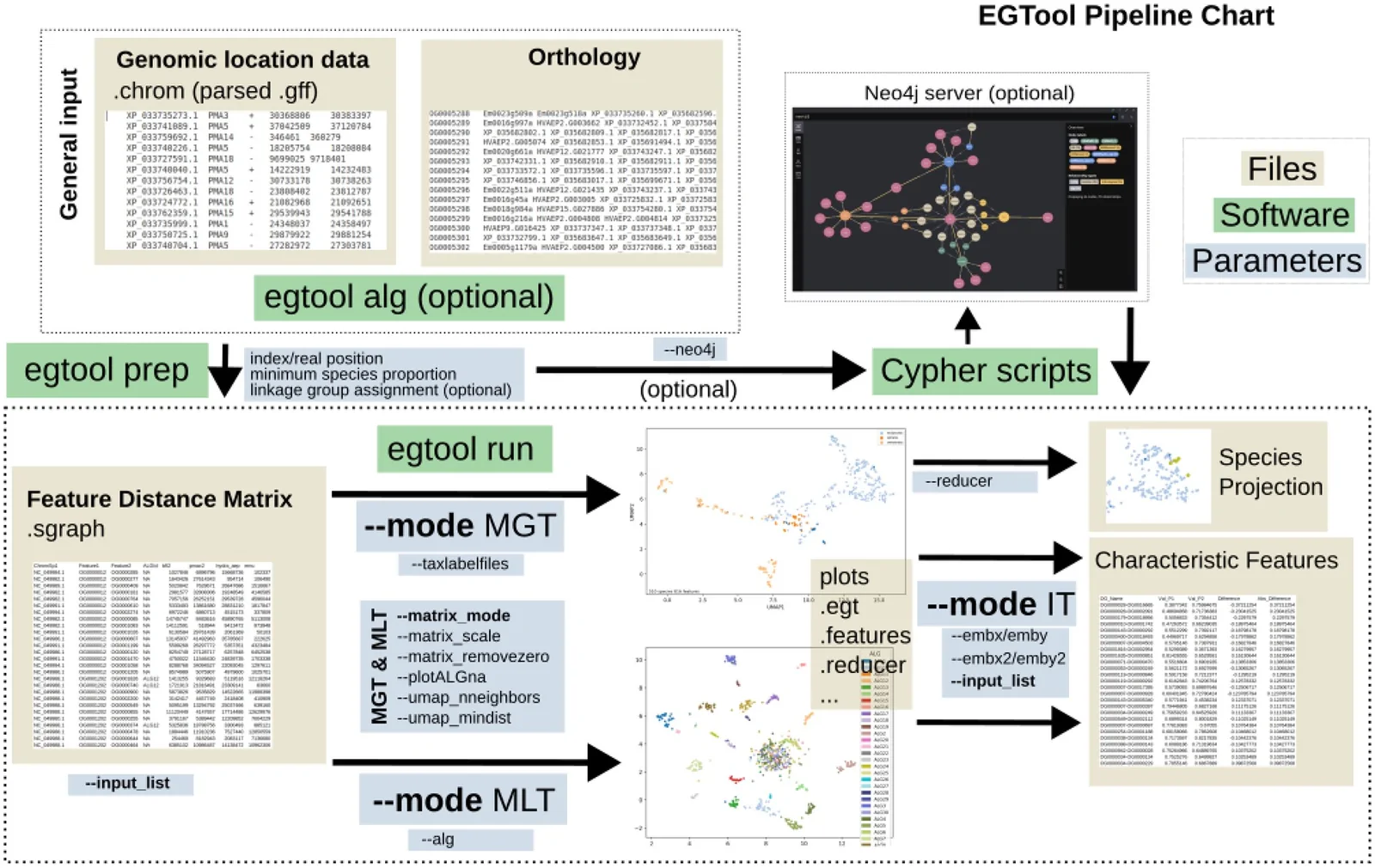
EGTool workflow is structured into different data types, sources, software, and parameters. The egtool package has several options for initial data processing, including ancestral linkage group (“egtool alg”) construction, as well as export (“egtool prep”) into the feature distance matrix format for further EGTool processing or for import into a graph database, such as Neo4j, using the Cypher query language. Each dot/circle in Neo4j represents an orthologous entity (can be a coding gene, a noncoding element, a chromosome, etc.). The command “egtool run” constitutes the main pipeline enabling the two main modes (MGT and MLT), providing intermediate files including embedding transformation objects (.reducer) that can be used for downstream analyses, such as mapping of new genomes onto available manifold space or inverse transform and comparisons between positions in the manifold space. On MGT plots, each dot corresponds to a species and is used to study cross-species evolutionary trajectories, evaluate phylogenetic associations, and, using inverse transform, calculate pairwise ortholog distance representation for any given point in the UMAP space. MGT dots can be colored by a variety of parameters, including taxonomic group, genome size, etc. MLT shows orthogroup associations, with each orthogroup (e.g. coding gene) represented as a dot; in this approach, dots can be colored based on their ALG association. Proximity of dots on both MGT and MLT plots suggests closer genomic distance-relatedness between either species (MGT) or orthogroups (MLT). More distant species (MGT) or orthogroup (MLT) associations represent divergent trajectories that are separated by multiple putative FWM or within-chromosome “entanglement” events. All major commands and steps represented in this figure are executed as part of the tutorial script.

The example tutorial also includes step-by-step calculation of an .sgraph file from four .chrom files for four example species and OrthoFinder-derived orthologs, enabling the identification of the metazoan ALGs (Simakov *et al.* 2022). The prep_egt script can also export data for loading into a Neo4j graph database (https://neo4j.com/) for further exploration and analysis (Schultz *et al.* 2026). EGTool exports a file (.sgraph format) that encodes species, orthogroups, chromosomes, and optional ALG assignments as linked graph entities, enabling interactive exploration of conserved associations and genome topology relationships across datasets.

### 2.3. EGT analyses

EGTool has two major processing modes of .sgraph files: MGT and MLT (Schultz *et al.* 2026, Schultz and Simakov 2026, Fig. 1). After building the homology .sgraph file (with or without ALG annotation), EGTool calculates manifold projections and analyzes them (“egtool run” command). The tool has three mandatory parameters: --mode, --matrix_mode, and --input_list. An additional parameter, --taxlabelfiles, allows users to specify NCBI taxonomy IDs to label data on the manifold projection (Fig. 1).

In MGT mode, individual genomes are fit into an embedding based on pairwise distances between homologous genomic features. MGT mode is intended to identify groupings of species and evolutionary trajectories (Schultz *et al.* 2026).

In MLT mode, pairwise distances are averaged across genomes and then projected onto the manifold. MLT reveals conserved positional relationships among orthologous features within a given clade. Labeling points by ALG assignment shows which loci share an ancestral chromosomal location. Example data file mlt_test_sub.sgraph has four species from our previous publication represented (*Branchiostoma floridae*, *Pecten maximus*, *Hydra vulgaris* AEP, *Ephydatia muelleri*; Simakov *et al.* 2022) and the MLT plot of it identifies clusters that correspond to the individual ALG identities (Fig. 1). Users can further change basic UMAP parameters such as minimal distance and neighbor number as implemented in the umap-learn Python package. The user can also specify distance measures (such as --matrix_mode euclidean or --matrix_mode manhattan) or use the raw .sgraph data as a distance matrix itself (--matrix_mode precomputed). Some EGTool parameters accept lists of values and will iterate and produce outputs for each of them. For example, --umap_nneighbors 2,5 will run the analysis for 2 and 5 n_neighbors values specified in UMAP separately. As has been done in the original EGT data publication (Schultz *et al.* 2026), this is useful for validation purposes and parameter sweep testing (Figs 1–4, available as supplementary data at *Bioinformatics Advances* online). Further analyses, such as silhouette quantification, can be used to assess embedding stability across input values (Fig. 5, available as supplementary data at *Bioinformatics Advances* online).

EGTool also supports several genomic distance transformations. In MGT mode, matrix preprocessing is controlled by --matrix_log1p, --matrix_removezero, and --matrix_scale. When enabled, EGTool applies a log1p transformation, removes orthogroup-pair rows containing at least one zero distance, and divides the remaining matrix by its maximum value, respectively; these operations are applied in that order when activated all together. This is particularly useful for comparing genomes after lineage-specific genomic expansions, such as in the lungfish (Meyer *et al.* 2021).

MGT and MLT modes produce files in the ./out directory and include the manifold projection image (.png), tabular data of the projection (.egt), binary stored UMAP and feature vector (.reducer and .features), as well as the raw data that went into UMAP analysis (after any normalization, .egtin).

Once UMAP analysis has concluded, users can extract the features that contribute most to a particular region of the embedding using --mode IT (inverse transform). This mode requires the --reducer parameter to point to the .reducer file containing the UMAP transformation object and coordinates in the projection space (--embx and --emby). The output .tsv file will contain an ordered estimate of the contributions of genomic features (for MLT) or genomic feature pairs (for MGT) that are characteristic of the specified location in the manifold space. This is a particularly useful feature to identify genomic features and their propensity to contribute to specific ALGs (in the MLT mode) or genomic feature pairs that are preserved at consistent distances in one clade (in the MGT mode). If the user specifies an additional location on the UMAP (--embx2 and --emby2 parameters), EGTool will compute the difference between these two points, enabling identification of enriched features in one location of the manifold projection compared to another.

### 2.4. Interpretation of EGTool results and further processing

By profiling genomic distances across many genomes, EGTool can identify constrained and putatively regulatory-constrained genomic states (Schultz *et al.* 2026). EGTool both visualizes complex genomic architecture (Fig. 1) and exports characteristic markers (.tsv output of the --mode IT as well as .egt output of both --mode MLT and --mode MGT). Because the tool is agnostic to the type of input features, alternative distance data [such as 3D contact frequencies from chromosome conformation capture, e.g. Lieberman-Aiden *et al.* (2009) and Schultz and Simakov (2026)] can be analyzed in a similar framework. Feature pair outputs can further define clade-specific phylogenetic markers, including chromosomal-level FWM characters (Simakov *et al.* 2020, 2022; Schultz *et al.* 2023) as well as putative entangled locally mixed loci (Simakov and Wagner 2025, Rogers *et al.* 2026, Schultz *et al.* 2026). As with other comparative genomics approaches, EGTool results depend on assembly quality, orthology inference, chromosome completeness, and user-defined parameter settings. In addition, because UMAP is a stochastic manifold-learning method, the resulting embeddings should be interpreted as exploratory summaries of genomic configurations that help identify additional phylogenetic signals. For further stability testing of the results, users can test different seed parameter values (Fig. 6, available as supplementary data at *Bioinformatics Advances* online) and orthogroup or species sampling (Fig. 7, available as supplementary data at *Bioinformatics Advances* online).

### 2.5. Species projection onto existing manifolds

A key feature of EGTool is the ability to project new genomes onto a previously computed manifold without refitting the UMAP model. MGT mode supports mapping of new genomic data onto an existing manifold via the --reducer option, which points to a previously computed UMAP reducer object. EGTool aligns the new genomes’ features to match the original model and projects them onto the existing embedding without refitting, supporting classification and phylogenetic-like analysis of genomes in terms of their genomic distances and configurations. An example command is included in the tutorial.

### 2.6. Performance

Following orthology inference, the complete EGTool tutorial pipeline comprising ALG detection, matrix preparation, and multiple MGT, MLT, and inverse transform analyses across the example dataset of 510 metazoan genomes and 3113 distances of orthologous locus pairs (1274 loci) completes in 14-30 min on a cluster or a mid-spec laptop. Individual steps scale with dataset complexity. ALG detection and distance preparation for four species complete in under 2 min, MLT analysis of 1025 orthogroups in precomputed mode runs in approximately 4 min, and MGT projection of 510 genomes completes in under 9 min. Mapping additional genomes onto an existing manifold is efficient, requiring <10 s for six new species. These runtimes make it easy to explore parameter settings iteratively, such as distance transformations, UMAP parameters, and ALG subsets. EGTool has been tested on an ASUS ExpertBook laptop running Linux Mint, an Apple MacBook Pro with an M1 Pro processor running macOS Sonoma 14.5, the Life Science Compute Cluster (LiSC) at the University of Vienna running Rocky Linux 9.6, and the Sherlock high-performance computing cluster at Stanford University running CentOS 7.

## 3 Example

The EGTool distribution includes a tutorial that demonstrates an end-to-end analysis, from the definition of ALGs and preparation of genomic distance data to MLT and MGT analyses, projection of additional genomes, and inverse transformation of the resulting manifold. The tutorial initially uses four example species together with their chromosome annotation and orthogroup files. First, egtool alg identifies chromosomal homologies among the four genomes using the ALG detection method. In this example, ALG identifiers are assigned to supported chromosome-homology chains containing at least five shared orthogroups, and the resulting orthogroup-to-ALG assignments are written to an alg.list file.

The resulting ALG assignments are then supplied to egtool prep together with the orthogroup and .chrom files which are provided in the test_input directory to construct an .sgraph genomic distance matrix. The example uses real base-pair coordinates and permits at most two paralogous representatives of an orthogroup per species. A pair of orthogroups is retained only when both are on the same chromosome in all four genomes. For each retained orthogroup pair, the preparation step calculates the genomic distance between the corresponding features (in this example between genes) in each species, and associates the orthologous feature pair with the available ALG annotation. This produces data.sgraph, which is then used directly in the following topology analyses.

The tutorial next shows a few example runs of MLT and MGT modes. MLT analysis examples show precomputed or Euclidean matrix modes by either excluding the orthologs without ALG assignment, or by subsetting the results only to the orthologs that are members of selected ALGs. MLT shows orthologous features as dots and visually distinguishes features that come from different chromosomes or ALGs. Focusing on specific ALGs or chromosomes enables visualization of the embedding structure within, highlighting constraints on feature movement within a chromosome.

For MGT mode, a large .sgraph dataset containing 510 metazoan genomes is used, which is also provided in the EGTool distribution. Taxonomy files for three clades are also available to color the dots representing the species from these clades in the resulting MGT plot. The first MGT command uses the default matrix representation, while another command shows how to remove feature pair rows containing zero distances and how to scale the distance matrix. Coloring dots (species) by their taxonomic group shows clear separation between major clades.

Finally, the tutorial demonstrates two downstream uses of a fitted MGT model. First, a second .sgraph dataset containing additional genomes (species) is projected onto this precalculated manifold, allowing new genomes to be placed onto existing manifolds, thus aiding classification without having to rerun the complete analysis. Second, the IT (inverse transform) mode is applied to a pair of selected coordinates in the MGT embedding. IT reconstructs an estimated orthogroup-pair distance profile for each selected position in the MGT embedding. In the two-point example, EGTool calculates the difference between the two reconstructed profiles and ranks orthogroup pairs according to the absolute magnitude of this difference, highlighting genomic distance features that most strongly distinguish the selected embedding positions.

## 4 Conclusion

EGTool provides an accessible, end-to-end pipeline for applying topological approaches to multiscale genomics. Its modular design, support for diverse distance metrics and input formats, and ability to project new genomes onto existing manifolds make it well suited for iterative exploration of genome architecture across large phylogenetic datasets. By combining dimensionality reduction with inverse transform analysis, the tool enables the generation of testable hypotheses of characteristic features that define chromosomes and genomes in evolution.

## Supplementary Material

vbag330_Supplementary_Data

## Data Availability

The data underlying this article are available at https://bitbucket.org/viemet/egtool/src/main/
