# Supplementary material for: EGTool: an evolutionary genome topology tool for multiscale comparative genomics
Source: Bioinform Adv. 2026 Nov 2;6(1):vbag330. doi: 10.1093/bioadv/vbag330 (PMC13770478; doi:10.1093/bioadv/vbag330)
Supplement: Supplementary file 1 [file vbag330_supplementary_data.pdf]

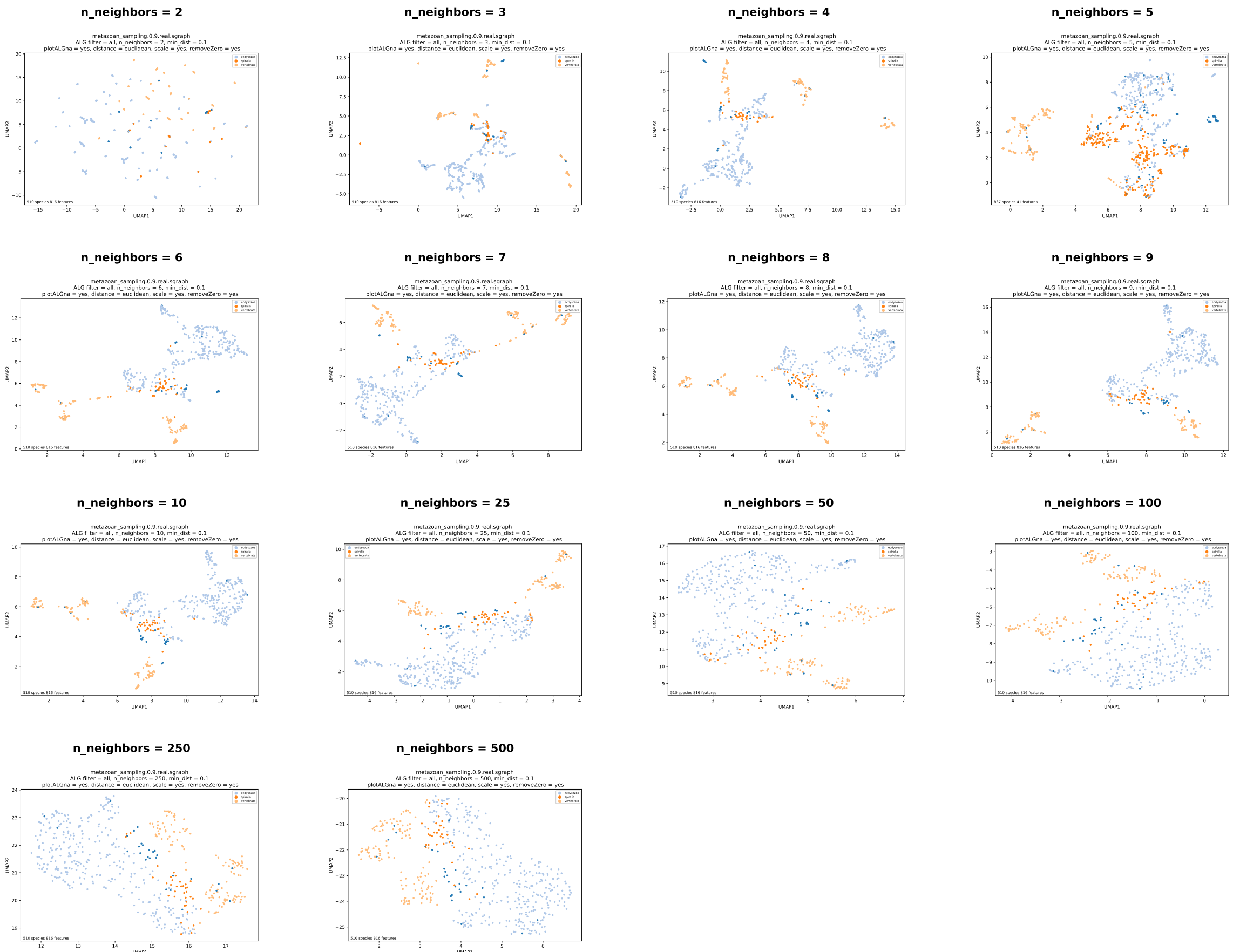

Supplementary Figure S1. Effect of UMAP n_neighbors with scaling enabled. UMAP embeddings generated using different n_neighbors values (2, 3, 4, 5, 6, 7, 8, 9, 10, 25, 50, 100, 250, and 500), while all other settings were kept constant (min_dist = 0.1, scaling = yes, log1p = yes, removezero = yes). Points represent species and are colored according to their assigned clade.

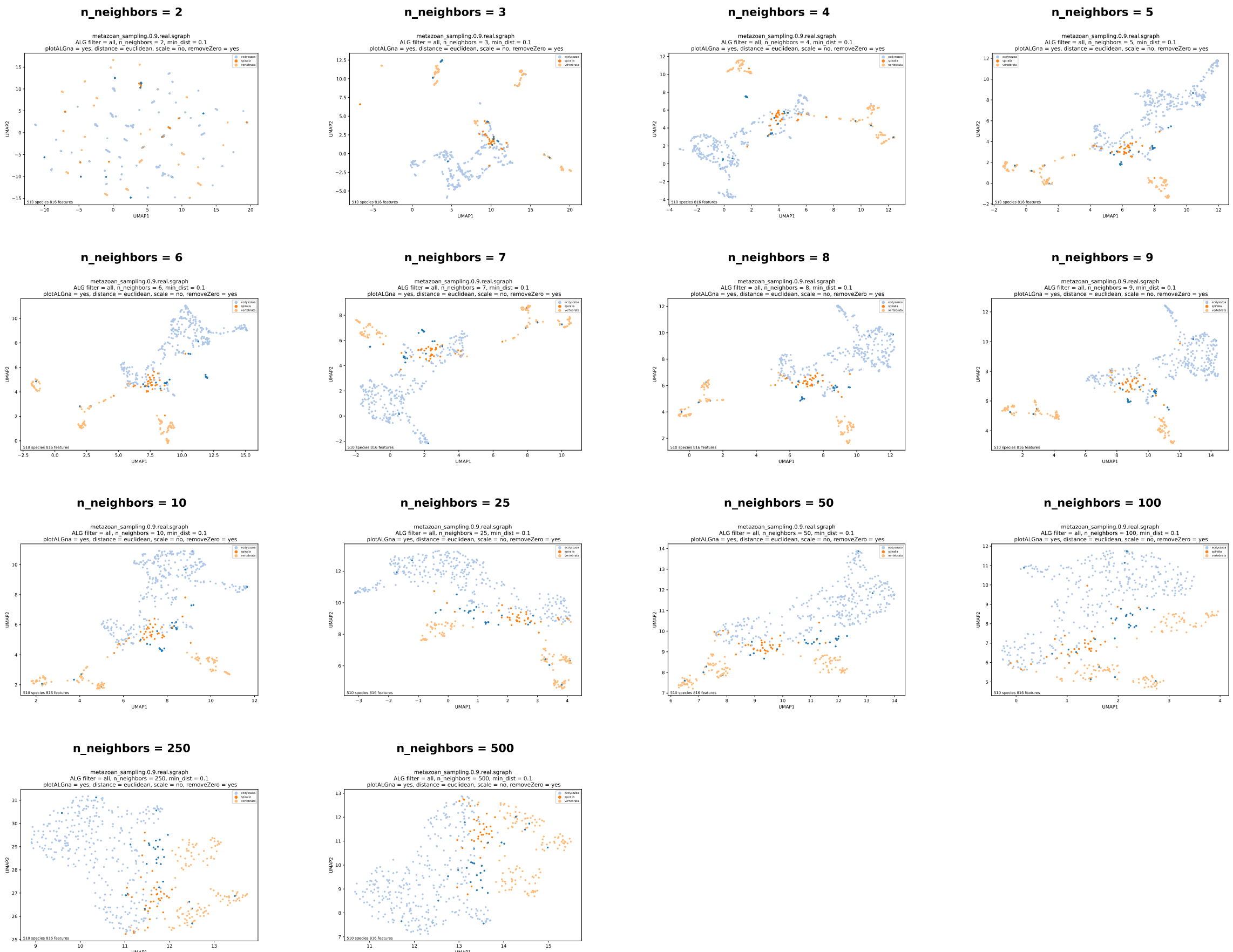

Supplementary Figure S2. Effect of UMAP n_neighbors without scaling. UMAP embeddings generated using different n_neighbors values (2, 3, 4, 5, 6, 7, 8, 9, 10, 25, 50, 100, 250, and 500), while all other settings were kept constant (min_dist = 0.1, scaling = no, log1p = yes, removezero = yes). Points represent species and are colored according to their assigned clade.

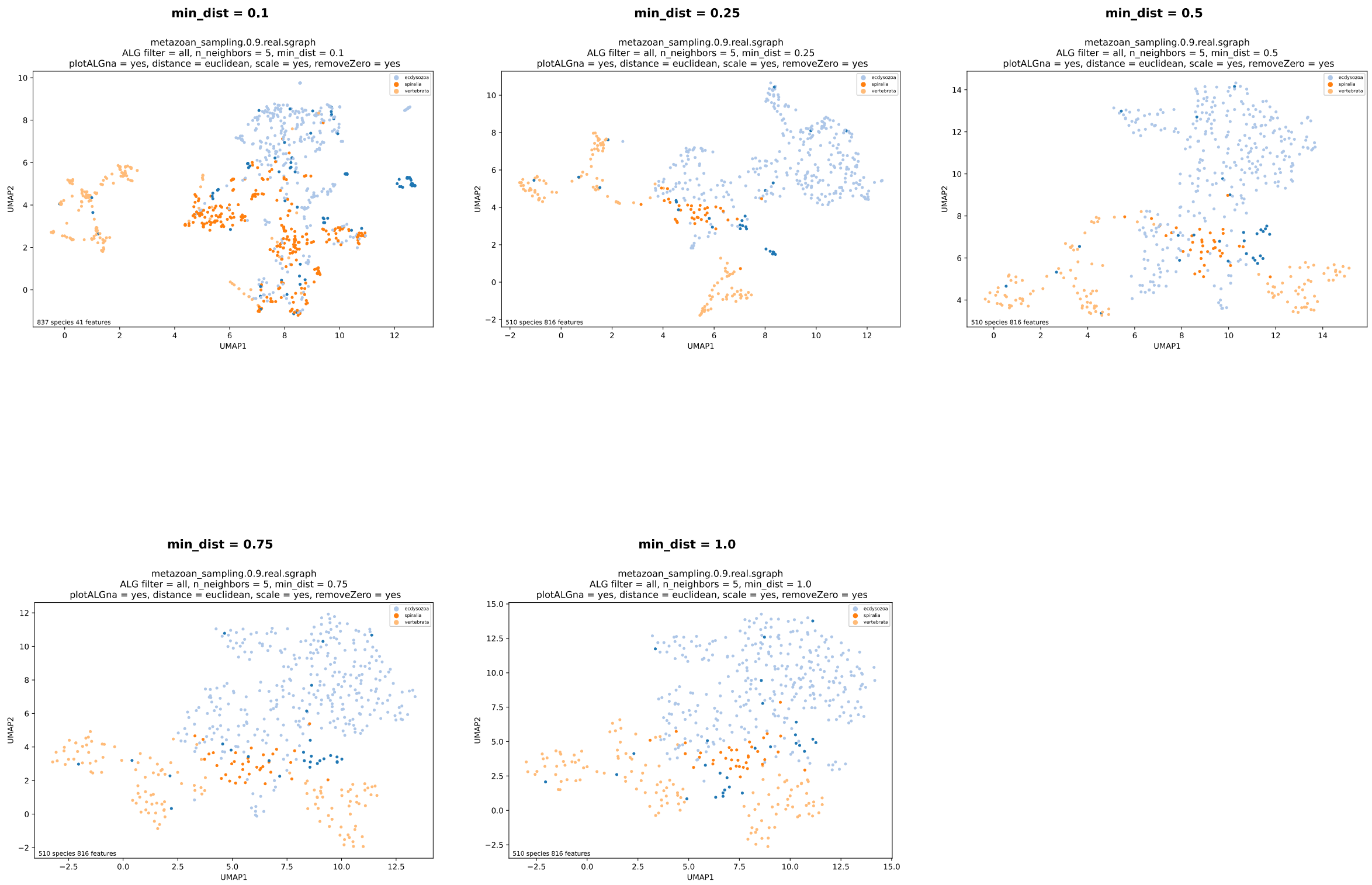

Supplementary Figure S3. Effect of UMAP min_dist with log transformation enabled. UMAP embeddings generated using min_dist values of 0.1, 0.25, 0.5, 0.75, and 1.0, while all other settings were kept constant (n_neighbors = 5, scaling = yes, log1p = yes, removezero = yes). Points represent species and are colored according to their assigned clade.

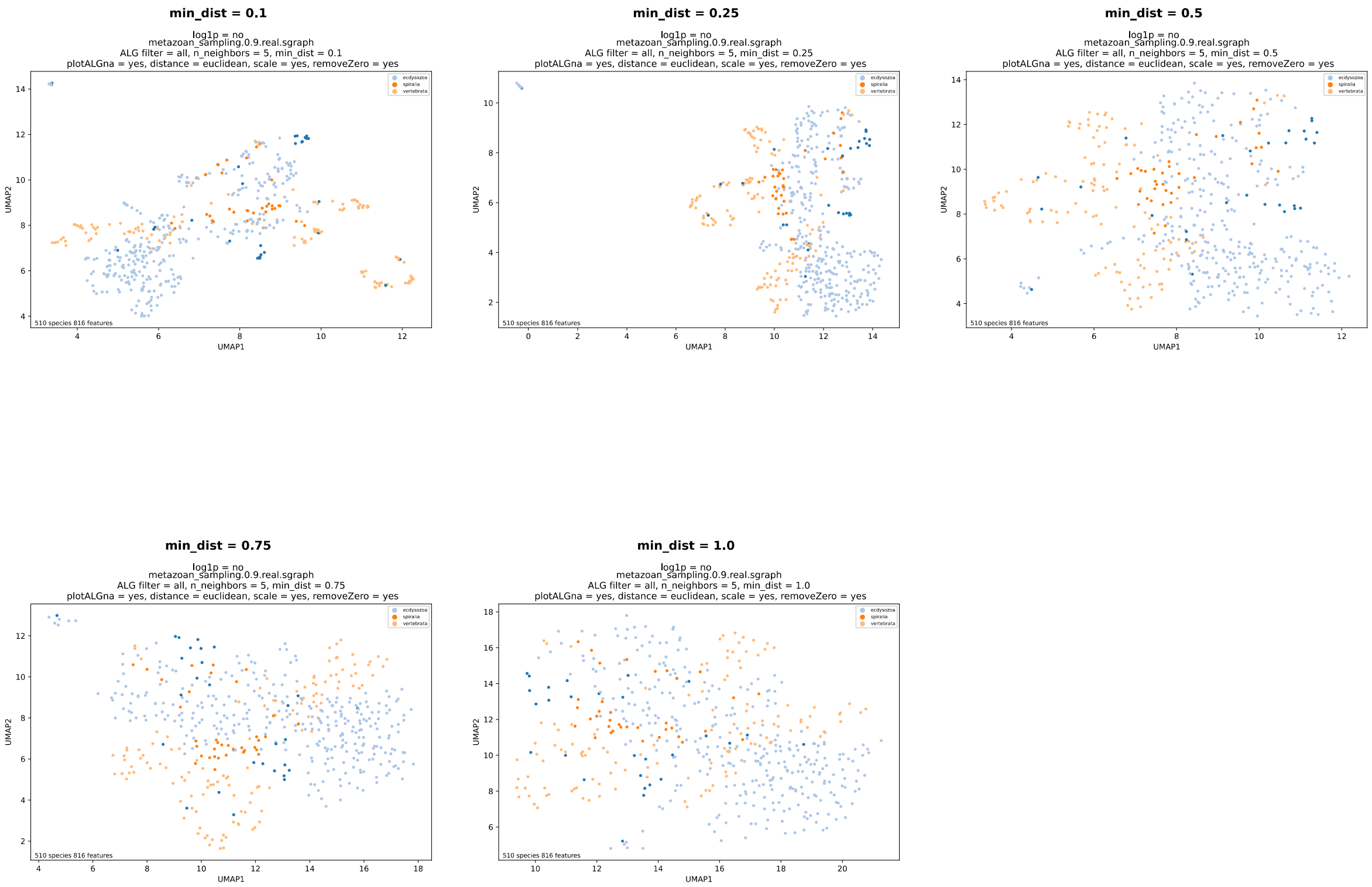

Supplementary Figure S4. Effect of UMAP min_dist without log transformation. UMAP embeddings generated using min_dist values of 0.1, 0.25, 0.5, 0.75, and 1.0, while all other settings were kept constant (n_neighbors = 5, scaling = yes, log1p = no, removezero = yes). Points represent species and are colored according to their assigned clade.

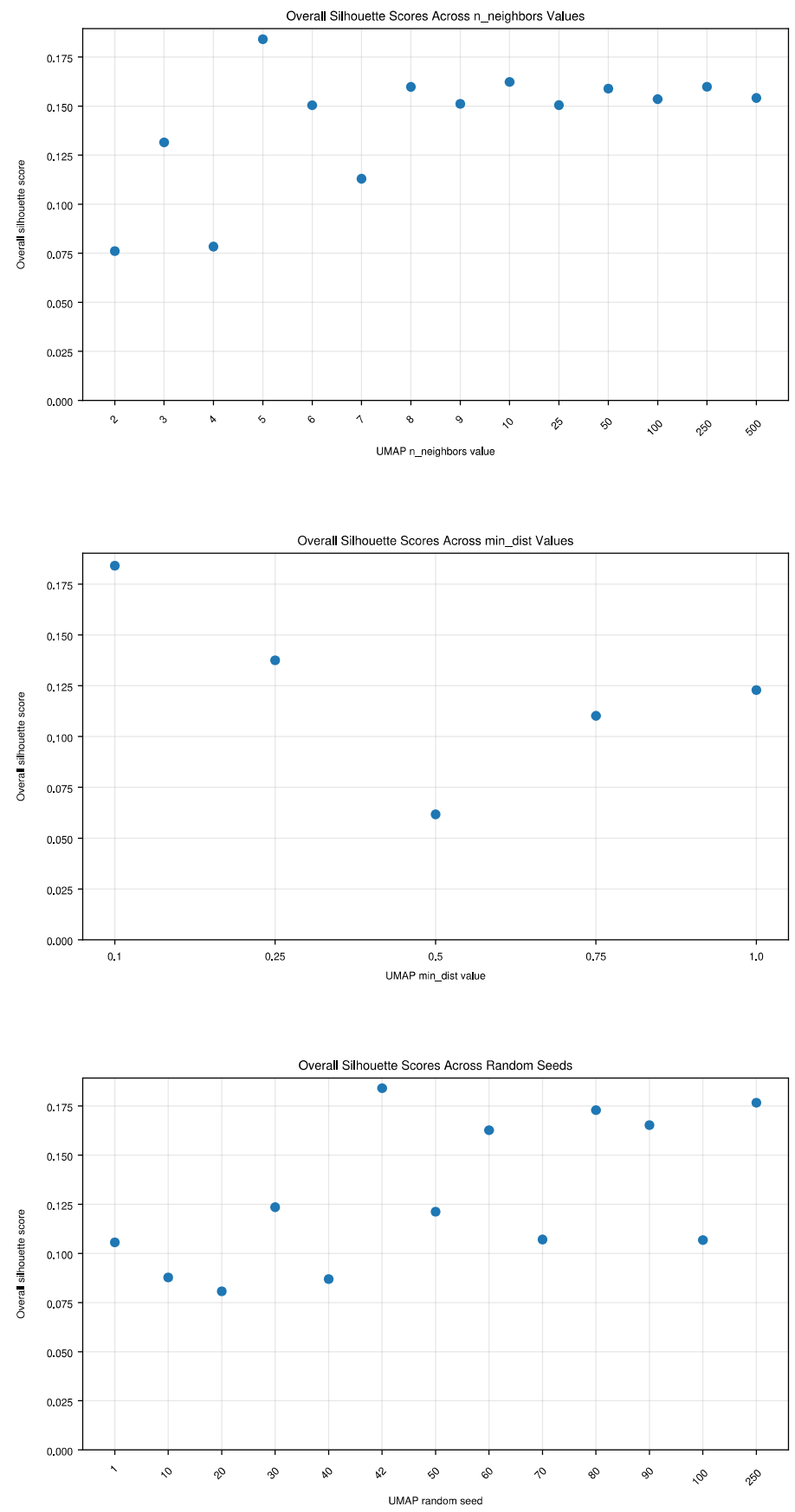

Supplementary Figure S5. Silhouette scores across UMAP parameter sweeps. Overall silhouette scores were calculated for UMAP embeddings generated with different n_neighbors values, min_dist values, and random seeds from the two-dimensional UMAP coordinates with sklearn.metrics.silhouette_score using predefined clade assignments as group labels. Silhouette scores remained positive across all tested parameter settings and showed broadly comparable values across each sweep, showing that the overall clade pattern remained similar. This supports the robustness of the observed genomic patterns to parameter variation and stochastic initialization.

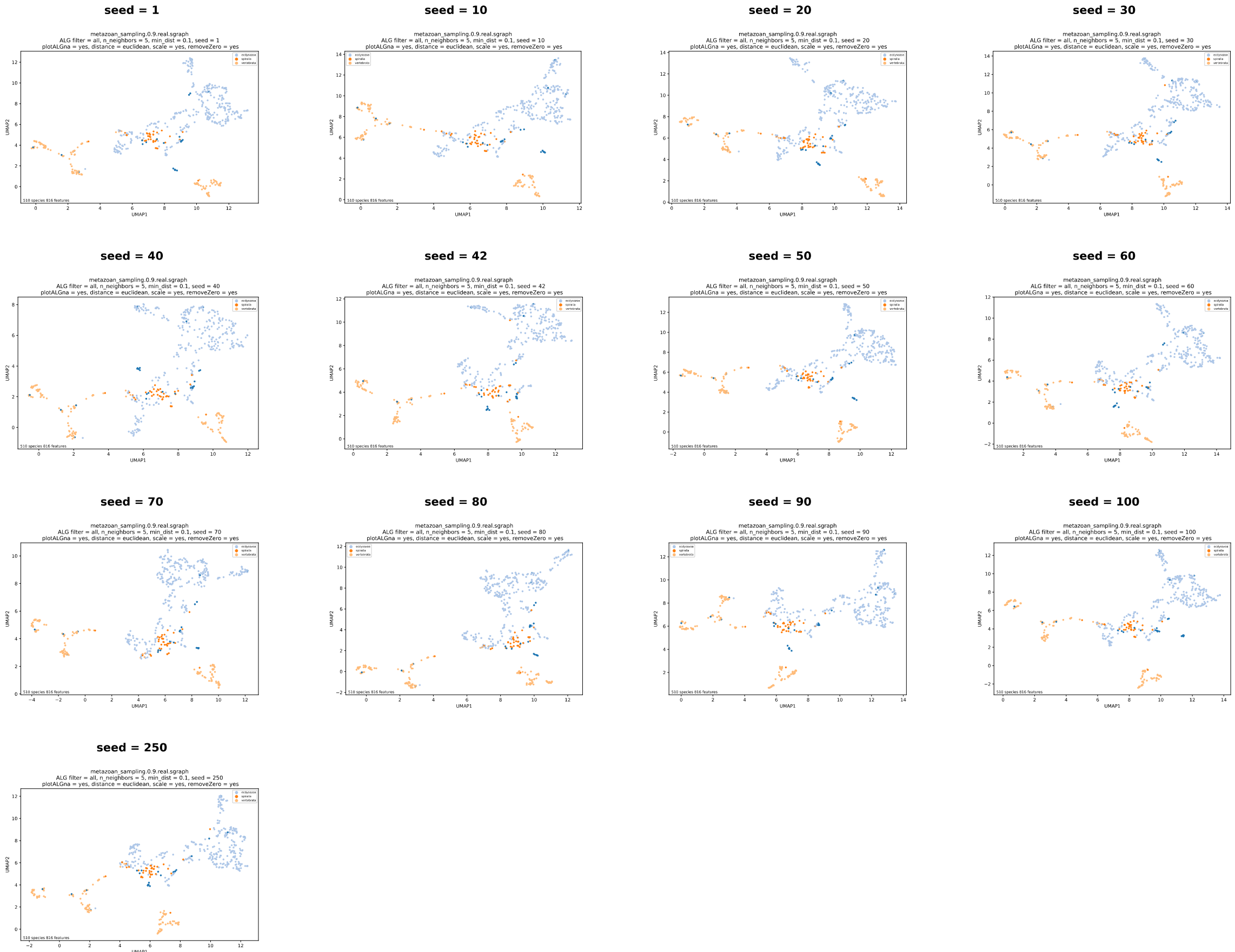

Supplementary Figure S6. Effect of UMAP random initialization. UMAP embeddings generated using 13 different random seeds (1, 10, 20, 30, 40, 42, 50, 60, 70, 80, 90, 100, and 250), while all other settings were kept constant (n_neighbors = 5, min_dist = 0.1, scaling = yes, log1p = yes, removezero = yes). Points represent species and are colored according to their assigned clade.

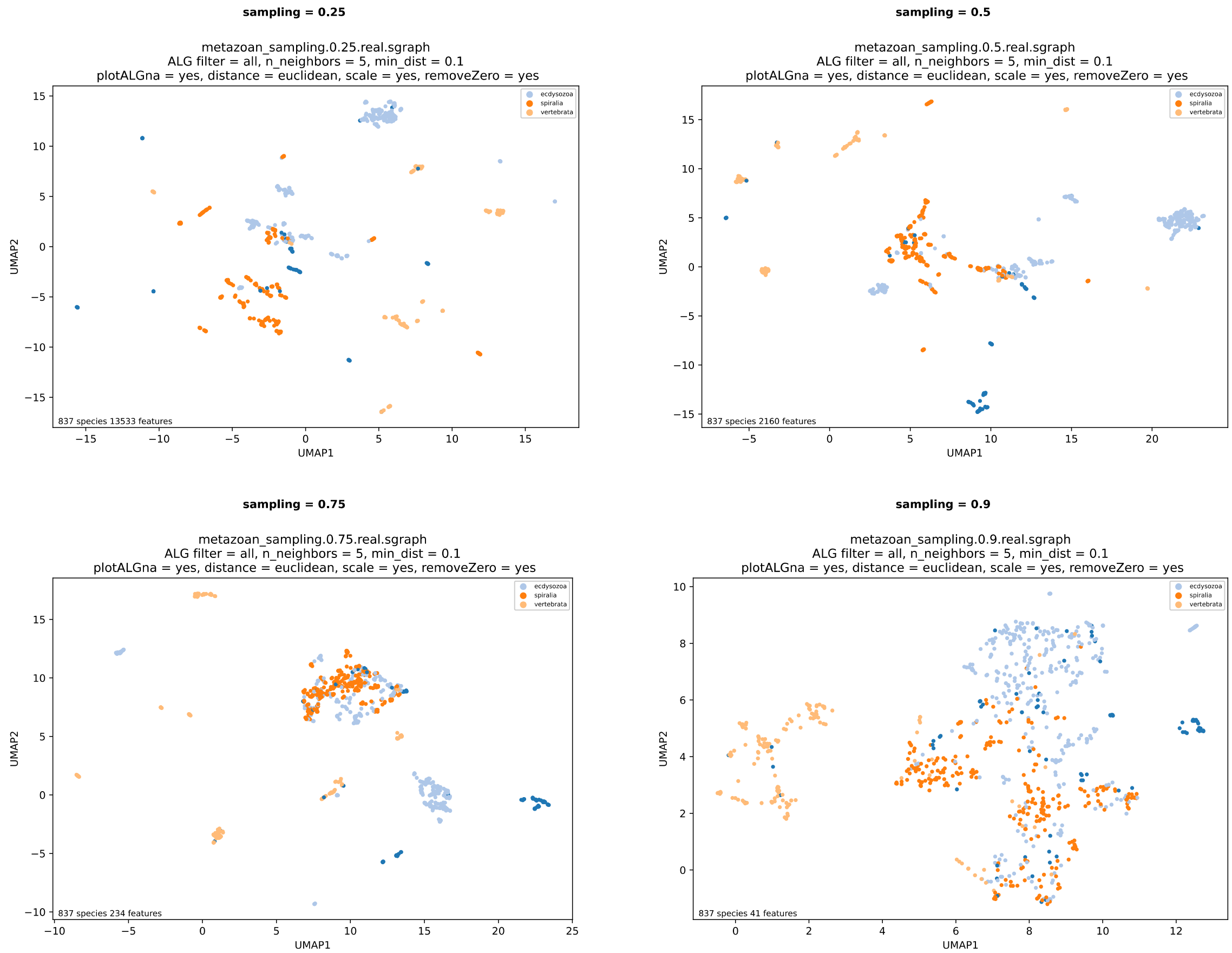

Supplementary Figure S7. Effect of the input sampling parameter on the UMAP embedding. UMAP embeddings generated using sampling values of 0.25, 0.5, 0.75, and 0.9 (at least 25%, 50%, 75% and 90% orthogroup pair representation across species, respectively), while the UMAP and preprocessing settings were kept constant (n_neighbors = 5, min_dist = 0.1, scaling = yes, log1p = yes, removezero = yes). Points represent species and are colored according to their assigned clade.
